# Efficacy and Safety of Desvenlafaxine for the Treatment of Persistent Idiopathic Dentoalveolar Pain: A Retrospective Observational Study

**DOI:** 10.1155/prm/8835808

**Published:** 2026-05-17

**Authors:** Minying Liu, Hao Ren, Chunmei Zhao, Lan Meng, Ying Shen, Fang Luo

**Affiliations:** ^1^ Department of Pain Management, Beijing Tiantan Hospital, Capital Medical University, Beijing, China, ccmu.edu.cn

**Keywords:** desvenlafaxine, effectiveness, persistent idiopathic dentoalveolar pain, serotonin and norepinephrine reuptake inhibition

## Abstract

**Background:**

Previous studies on serotonin and norepinephrine reuptake inhibitors (SNRIs) such as duloxetine and venlafaxine have demonstrated efficacy in treating persistent idiopathic dentoalveolar pain (PIDP). However, a third‐generation SNRI with favorable tolerability remains understudied in treating PIDP. The study aims to investigate the role of desvenlafaxine in improving PIDP.

**Methods:**

Clinical data for patients diagnosed with PIDP who received desvenlafaxine treatment (50–150 mg per day) from March 2024 to January 2025 were retrospectively reviewed at our hospital. Through systematic review of electronic medical records, we extracted demographic and baseline pain characteristics, along with the effectiveness and safety of desvenlafaxine during a 3‐month follow‐up assessed by the Numeric Rating Scale‐11 (NRS‐11) scores for pain and adverse effects. Binary logistic regression was used to identify predictors of response.

**Results:**

Among 103 patients, pain relief was achieved in 90 patients (87.4%), as evidenced by a ≥ 50% reduction in NRS‐11 scores (*p* < 0.001), while the remaining 13 patients (12.6%) discontinued medications due to insufficient therapeutic efficacy. Binary logistic regression analysis identified shorter pain duration as an independent predictor of a favorable response to desvenlafaxine, with an odds ratio of 1.037 (95% CI = 1.016–1.058, *p* < 0.001). Adverse events were reported in 15 patients (14.6%), all mild and transient.

**Conclusions:**

The administration of desvenlafaxine serves as an effective and safe therapeutic approach for patients with PIDP. Patients with a short history of PIDP are more likely to benefit from desvenlafaxine treatment.

## 1. Introduction

Persistent idiopathic dentoalveolar pain (PIDP), formerly termed atypical odontalgia, is a debilitating chronic unilateral intraoral dentoalveolar pain disorder characterized by persistent, deep, and dull pain without detectable pathology, recurring daily for over 2 h for more than 3 months [1, 2]. Typically presenting as localized, mild‐to‐moderate pain [1], PIDP often involves somatosensory abnormalities [3]. Its prevalence remains uncertain, though it predominantly affects women with a mean age of 51.1 years and frequently follows dental procedures [4–6].

The pathophysiology of PIDP is not fully understood, with psychogenic, vascular, neuropathic, or idiopathic origins. The most accepted hypothesis is that PIDP is a neuropathological condition, often linked to deafferentation of afferent trigeminal fibers from dental procedures like root canals or extractions [7, 8].

A poor grasp of the underlying pathophysiological mechanisms of PIDP is a significant factor contributing to management challenges. Current PIDP management involves topical agents such as local anesthesia, capsaicin, or botulinum toxin, and/or systemic treatment with tricyclic antidepressants (TCAs), opioids, or selective serotonin–norepinephrine reuptake inhibitors (SNRIs), often based on expert opinions. Topical agents may offer incomplete relief or cause side effects [9]. Among systemic options, the analgesic effect of TCAs is primarily attributed to the capacity to block the reuptake of serotonin and norepinephrine, key neurotransmitters in the pain inhibitory system [10]. However, TCAs often have insufficient efficacy and nearly half of patients report side effects [11]. Opioids are generally not recommended, as the risks of hyperalgesia, tolerance, and dependence outweigh any potential benefit [12, 13]. Venlafaxine and duloxetine, both SNRIs, have demonstrated superior efficacy to TCAs in PIDP [14, 15]. Venlafaxine showed a 50% pain reduction in 80.6% of patients [14], while duloxetine provided pain relief in 77.0% [15]. However, some patients did not respond adequately or experienced intolerable side effects (3.1% discontinuing venlafaxine and 9.6% discontinuing duloxetine) [14, 15], highlighting the need for alternative SNRIs.

Desvenlafaxine, an active metabolite of venlafaxine approved in 2008 [16], has limited research in PIDP. A noninferiority trial suggested that desvenlafaxine may offer comparable efficacy to duloxetine in pain reduction for major depression with fewer adverse effects like nausea and dizziness [17]. Additionally, it has shown a lower incidence of adverse effects compared to duloxetine [18] and less nausea than venlafaxine [19]. Moreover, desvenlafaxine does not appear to affect the effectiveness of hypotensive drugs, in contrast to duloxetine and venlafaxine [20]. Current evidence suggests desvenlafaxine may offer comparable efficacy yet a potentially safer treatment for PIDP. Therefore, we retrospectively analyzed the clinical data of patients diagnosed with PIDP to explore the effectiveness and safety of desvenlafaxine.

## 2. Methods

This retrospective, single‐center observational study was performed at our hospital, adhering to the principles of the Declaration of Helsinki. The study was approved by the Institutional Review Board of the hospital (Approval Number: KY2026‐022‐02). Informed consent was obtained from all patients before inclusion in the study, and each participant was thoroughly informed that their involvement would be entirely voluntary and that they retained the right to withdraw from the study at any time.

The study posed minimal risk to patients, and all analyzed data were deidentified. Retrospective medical records of patients diagnosed with PIDP who received desvenlafaxine treatment at the hospital between March 2024 and January 2025 were reviewed.

### 2.1. Inclusion Criteria

1. Patients were diagnosed with PIDP according to the ICOP‐1 criteria [1] by associate chief pain physicians or chief pain physicians;
2. Patients were treated with desvenlafaxine;
3. Patients aged over 18 years;
4. Follow‐up data were available for at least 3 months, unless patients discontinued taking desvenlafaxine because of side effects or poor efficacy.

### 2.2. Exclusion Criteria

1. Concurrent use of other medications or physical therapy for pain during the treatment with desvenlafaxine;
2. Presence of diabetic neuropathy;
3. History of psychiatric illness or substance abuse;
4. Concomitant presence of other painful medical conditions other than PIDP.

### 2.3. Medication Regimen

All patients adhered to a standardized dosing regimen of desvenlafaxine (Desvenlafaxine Succinate Sustained‐Release Tablets, CSPC Ouyi Pharmaceutical Co., Ltd) at our hospital. Desvenlafaxine was initiated at 50 mg per day, to be taken with breakfast. If pain relief was insufficient and the drug was well tolerated, the dose could theoretically be further escalated by 50 mg per day after 3 days, up to a maximum of 400 mg per day after 7 days, according to the product labeling and previous studies [19, 21]. However, in routine clinical practice, dose adjustment was individualized based on pain relief, patient tolerance, and willingness to continue dose escalation. The dose of desvenlafaxine was maintained once the patient’s pain was either completely relieved or reduced to a level deemed satisfactory by the patient, who declined further dose escalation. Once intolerable side effects emerged as the dose increased, the dose was reduced to the last tolerable dose. Participants were managed through routine outpatient visits every 2 weeks during the first month of treatment, followed by monthly visits thereafter. All clinical evaluations, including efficacy assessment, adverse events documentation, dose adjustment, and prescription issuance, were conducted at the outpatient department. In addition, patients were instructed to routinely monitor their blood pressure and heart rate after starting desvenlafaxine.

### 2.4. Data Collection and Measurement

Demographic characteristics (age and gender), comorbidities, pain duration, pain laterality, and pain intensity assessed by Numeric Rating Scale‐11 (NRS‐11) scores, previous medications, dental procedure history, and symptoms of depression assessed by the Hamilton Depression Rating Scale (HDRS) scores were extracted from electronic records. The NRS‐11 score is a validated scale to assess pain intensity and consists of 0–10 points, with 0 indicating no pain and 10 indicating the worst pain imaginable [22]. The HDRS is a 17‐item scale used to assess depressive symptoms [23]. Each item is scored on a scale of 0 (*not present*) to 4 (*severe*), with some items scored from 0 to 2. The total score ranges from 0 to 54, with higher scores indicating greater depression levels. The severity ranges for the HDRS are as follows: *no depression* (0–7), *mild depression* (8–16), *moderate depression* (17–23), and *severe depression* (≥ 24).

To improve the quality of medical care, we routinely assess the efficacy and side effects of drug use and record them in the outpatient medical system. We collected data on the effectiveness and safety of desvenlafaxine, which included the following variables: treatment onset time, NRS‐11 and HDRS scores in each visit time, current dose of desvenlafaxine, and adverse effects, along with subsequent treatment. The time to a 30% reduction in the NRS‐11 score from baseline was defined as treatment onset time. Treatment response was defined as a ≥ 50% reduction in the baseline NRS‐11 score, in line with previous retrospective studies of PIDP that used a ≥ 50% reduction in pain scores as the response criterion [14, 15]. Based on that, we divided subjects into responsive and nonresponsive groups. Adverse effects included nausea, vomiting, dry mouth, drowsiness, insomnia, dizziness, headache, fatigue, tachycardia, etc. The incidence of these adverse effects and the reason for discontinuing medication were collected.

### 2.5. Statistical Analysis

The data were analyzed using IBM SPSS Statistics Version 27.0. Categorical variables were presented as numbers and percentages, with analyses conducted via the chi‐square test or Fisher’s exact test. Continuous variables were reported as medians and interquartile ranges for non‐normally distributed data and as means and standard deviations for normally distributed data. For comparisons, analysis of variance was applied to normally distributed data, while the Kruskal–Wallis test was used for non‐normally distributed data.

For responsive patients, paired Wilcoxon tests were performed to compare their NRS‐11 scores at 2 weeks, 1, 2, and 3 months following treatment initiation with their baseline NRS‐11 scores. A *p* value of < 0.05 was considered statistically significant. The incidence of adverse effects was also detailed.

Factors linked to treatment response were assessed through binary logistic regression, presenting the adjusted odds ratio alongside 95% confidence intervals. The analysis considered baseline demographic and clinical characteristics, including age, sex, comorbidity, pain duration, pain laterality, baseline NRS‐11 score, previous medication use, history of dental procedures, and baseline HDRS score. Given the retrospective design and limited sample size, this regression analysis was considered exploratory, and the findings should be interpreted with caution.

## 3. Results

### 3.1. Patient Characteristics

**FIGURE 1:**
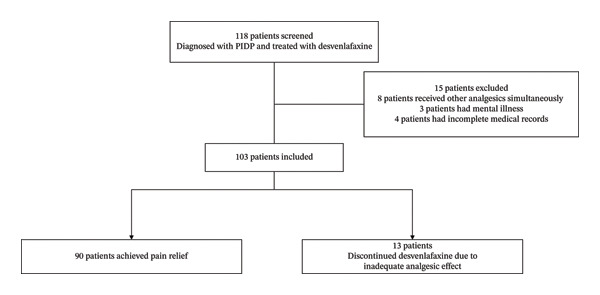
The flowchart of patient inclusion and exclusion.

From March 2024 to January 2025, a total of 118 patients diagnosed with PIDP were administered desvenlafaxine for pain management at our hospital’s pain clinic. Of these patients, 8 had concurrent use of other analgesics, 3 had psychiatric disorders, and 4 had incomplete medical documentation. Ultimately, 103 patients were eligible for inclusion (Figure 1). Prior to starting desvenlafaxine, patients had been undergoing treatments with medications such as nonsteroidal anti‐inflammatory drugs, antiepileptics, muscle relaxants, aminophenol oxycodone, or tramadol, yet they experienced inadequate pain relief.

In addition, 67 patients (65.0%) underwent dental treatment, such as tooth extraction, following a dental misdiagnosis that eventually had no effect on their pain or worsened their pain. Table 1 summarizes the demographics and pain‐related baseline data of included patients.

**TABLE 1:** Demographics and pain‐related baseline data of included patients (*n* = 103).

Variables	Values
Age, median (IQR), years	53 (40, 63)
Gender, no. (%)	
Male	22 (25.2)
Female	77 (74.8)
Comorbidity, no. (%)	31 (30.1)
Hypertension	7 (6.8)
Diabetes	14 (13.6)
Coronary heart disease	10 (9.7)
Pain duration, median (IQR), months	24 (6, 48)
Pain laterality, no. (%)	
Left side	38 (36.9)
Right side	39 (37.9)
Bilateral side	26 (25.2)
Numeric Rating Scale‐11 score, mean ± SD	7.50 ± 0.91
Previous medication use, no. (%)	98 (95.1)
NSAIDs	62 (60.2)
Antiepileptics	61 (59.2)
Muscle relaxants	14 (13.6)
Aminophenol oxycodone	12 (11.7)
Tramadol	3 (2.9)
History of dental procedures for treatment, no. (%)	67 (65.0)
HDRS score, median (IQR)	13 (6, 15)
Low score group ≤ 7, no. (%)	27 (26.2)
High score group ≥ 8, no. (%)	76 (73.8)

*Note:* NSAIDs, nonsteroidal anti‐inflammatory drugs.

Abbreviations: HDRS, Hamilton Depression Rating Scale; IQR, interquartile range; SD, standard deviation.

### 3.2. Efficacy of Desvenlafaxine

Of the 103 patients in our study, none discontinued treatment due to side effects. Pain relief was achieved within 3 months in 90 patients (87.4%), whereas treatment was discontinued by 13 patients (12.6%) due to insufficient efficacy.

**FIGURE 2:**
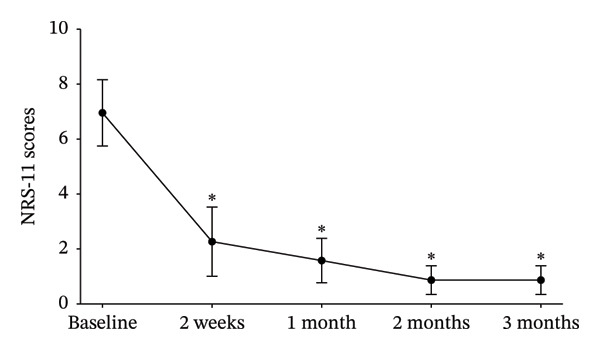
The time course of NRS‐11 in 90 patients who achieved pain relief. Each point represents the mean ± SD score. ^∗^Compared with baseline, *p* < 0.001, paired‐Wilcoxon tests. Abbreviation: NRS‐11: Numeric Rating Scale‐11.

The median daily dose at treatment onset was 50.0 mg (IQR: 50.0–50.0 mg) with a median time to treatment onset of 5 days (IQR: 2–7 days) from the initiation of treatment. For those who reported pain relief, the median dose at the time of relief was 50.0 mg per day (IQR: 50.0–100.0 mg per day). Significant reductions in NRS‐11 scores were observed at 2 weeks, 1 month, 2 months, and 3 months after treatment initiation, compared to baseline (*p* < 0.001, Figure 2). Patients who achieved pain relief continued with a median dose of 50.0 mg per day (IQR: 50.0–100.0 mg per day). In contrast, unresponsive patients were administered a maximum dose of 150.0 mg per day (IQR: 100.0, 150.0 mg per day).

### 3.3. Predictor of Efficacy

No statistically significant differences were identified between the two groups for variables such as age, gender, comorbidities, pain laterality, baseline NRS‐11 score, previous medication use, history of dental procedures, baseline HDRS scores for depression, and number of patients with HDRS ≤ 7 or ≥ 8 (Table 2). The pain duration was significantly longer in the nonresponsive group than in the responsive group (*p* < 0.001; Table 2). Binary logistic regression analysis indicated that shorter pain duration was an independent predictor of a favorable response to desvenlafaxine, with an odds ratio of 1.037 (95% CI = 1.016–1.058, *p* < 0.001).

**TABLE 2:** Comparison of the demographics and pain‐related baseline data between the responsive group and the nonresponsive group.

Variables	Responsive group (*n* = 90)	Nonresponsive group (*n* = 13)	*p* value
Age, median (IQR), years	53 (37, 63)	53 (45, 63)	0.588
Gender, no. (%)			0.846
Male	23 (25.6)	3 (23.1)	
Female	67 (74.4)	10 (76.9)	
Comorbidity, no. (%)	29 (32.2)	2 (15.4)	0.335
Hypertension	6 (6.7)	1 (7.7)	
Diabetes	13 (14.4)	1 (7.7)	
Coronary heart disease	10 (11.1)	0 (0.0)	
Pain duration, median (IQR), months	24 (6, 36)	72 (48, 72)	< 0.001
Pain laterality, no. (%)			0.194
Left side	34 (37.8)	4 (30.8)	
Right side	36 (40.0)	3 (23.1)	
Bilateral side	20 (22.2)	6 (46.2)	
Numeric Rating Scale‐11 score, mean ± SD	7.49 ± 0.95	7.62 ± 0.51	0.314
Previous medication use, No. (%)	88 (97.8)	13 (100.0)	0.762
NSAIDs	55 (61.1)	7 (53.8)	
Antiepileptics	53 (58.9)	8 (61.5)	
Muscle relaxants	12 (13.3)	2 (15.4)	
Aminophenol oxycodone	9 (10.0)	3 (23.1)	
Tramadol	3 (3.3)	0 (0.0)	
History of dental procedures for treatment, no. (%)	61 (67.8)	6 (46.2)	0.211
HDRS score, median (IQR)	12 (6, 14)	15 (10, 15)	0.082
Low score group, no. (%)	25 (27.8)	2 (15.4)	0.506
High score group, no. (%)	65 (72.2)	11 (84.6)	

*Note:* NSAIDS, nonsteroidal anti‐inflammatory drugs.

Abbreviations: HDRS, Hamilton Depression Rating Scale; IQR, interquartile range; SD, standard deviation.

### 3.4. Adverse Effects

A total of 15 patients (14.6%) experienced adverse effects associated with desvenlafaxine, the majority of which were relatively mild and transient. The most frequently reported adverse effect was nausea (Table 3). Most adverse effects occurred during the early phase of treatment and generally improved with rest or continued treatment at the current dose and were well tolerated. No patients reported significant changes in blood pressure and heart rate. All patients completed the treatment regimen. No discontinuations due to adverse effects were recorded.

**TABLE 3:** Adverse effects with desvenlafaxine (*n* = 103).

Adverse effects	Number of patients (percentage)
Nausea	12 (11.7)
Vomiting	7 (6.8)
Dry mouth	6 (5.8)
Drowsiness	3 (2.9)
Insomnia	5 (4.9)
Dizziness	6 (5.8)
Headache	5 (4.9)
Fatigue	3 (2.9)

## 4. Discussion

Our main finding is that 90 patients, comprising 87.4% of the cohort, continued desvenlafaxine treatment for at least 3 months with clinically meaningful pain relief. A striking 88.4% reduction in the mean NRS‐11 score was observed at 3 months compared with baseline. This high therapeutic efficacy and substantial decrease in pain intensity indicate that desvenlafaxine could provide a potential therapeutic benefit for patients with PIDP. Our findings could pave the way for new treatment protocols or the optimization of existing ones.

The reduction in pain scores observed with desvenlafaxine in PIDP patients in this study aligns with findings from previous studies on other SNRIs, such as duloxetine and venlafaxine. Duloxetine has been reported to achieve a pain relief rate of 77.0% [15], while venlafaxine reported a rate of 80.6% [14]. Additionally, a retrospective study on the administration of TCAs for atypical odontalgia suggested a pain relief rate of 65.9% [11]. Notably, the pain relief percentage observed with desvenlafaxine in our study exceeded these values, reaching 87.4%. These findings suggest that desvenlafaxine may offer superior efficacy within PIDP patients. However, prospective controlled studies are necessary to confirm these results and establish a more definitive comparison.

Binary logistic regression analysis revealed that a shorter pain duration is a significant predictor of responsiveness to desvenlafaxine in patients with PIDP. Findings comparable to those of venlafaxine reported by Xiao et al. [14] were also noted with duloxetine by Jia et al. [15] in analogous patients. Desvenlafaxine, similar to the commonly used venlafaxine and duloxetine, may exhibit greater efficacy when initiated in the early stages of PIDP. Consequently, we cautiously contend that patients with a shorter history of PIDP are more likely to benefit from desvenlafaxine treatment, while patients with a longer history of pain may need more aggressive treatment. No clinical or demographic variables significantly predicted treatment response to desvenlafaxine in our cohort. Although desvenlafaxine is primarily prescribed for depression, baseline HDRS scores did not predict treatment responsiveness, suggesting that the analgesic efficacy in PIDP may be mechanistically distinct from its antidepressant effects. This observation aligns with other SNRIs, such as venlafaxine [14] and duloxetine [15]. Further research into additional predictive factors is warranted to refine clinical approaches for implementing desvenlafaxine in the management of PIDP patients.

Patients who were taking other medications for pain during the 3 months of desvenlafaxine treatment were excluded. No analgesics were administered during the study, precisely to prevent any confounding effects on the outcomes. Following the initiation of desvenlafaxine treatment, pain relief was achieved within a median of 5 (IQR: 2–7) days, which was comparatively faster than that observed with venlafaxine (median: 9 days; IQR: 8–11 days) [14] and duloxetine (median: 5 days; IQR: 1–14 days) [15]. Nonetheless, desvenlafaxine generally takes up to a week to exert its effects, and a delayed response may occur in some patients. Participants should be advised to give the drug time to show its therapeutic effects, rather than hastily switching medications. Strategies to further shorten the time of treatment onset should also be explored in future studies.

The exact mechanism by which desvenlafaxine alleviates pain is yet to be determined, and its therapeutic action in PIDP is yet to be fully understood. As a SNRI, desvenlafaxine is known to increase the levels of serotonin and norepinephrine, which are involved in pain modulation through the activation of descending pain pathways [24]. In our cohort, among patients who achieved pain relief, the median dose at pain relief was 50.0 mg per day (IQR: 50.0–100.0 mg per day), while the initial dose of it was also 50 mg. In comparison, the initial dose of duloxetine was 20 mg per day, with the final median dose required for pain relief reaching 60 mg per day [15]. Similarly, the initial median dose of venlafaxine was 37.5 mg per day, increasing to a final dose of 112.5 mg per day [14]. The relatively low dose required in our cohort may reflect differences in study design, patient heterogeneity, and individualized dose titration in real‐world practice. Given the dosage form of the sustained‐release tablets and the few known side effects of desvenlafaxine, all patients took 1 desvenlafaxine tablet daily and did not initiate titration at lower doses. In addition, for most patients, the initial dose of desvenlafaxine was the dose for pain relief. Hence, adherence to medication may have been higher in these patients. Nevertheless, these observations should be interpreted cautiously and require confirmation in future prospective studies.

In this study, 14.6% of patients suffered from side effects, with nausea being the most frequently reported side effect. Fortunately, most cases suffered only from mild nausea. The overall rate of adverse effects (14.6%) was lower than that observed with other SNRIs, including venlafaxine (49.6%) [14] and duloxetine (17.8%), in patients with PIDP [15]. Furthermore, side effects were generally transient, occurring primarily at the onset of treatment. Patients could tolerate them without withdrawing from treatment. The discontinuation rate due to side effects was 0%, markedly lower than that reported with venlafaxine (3.1%) [14] and duloxetine (9.6%) [15] in similar patient cohorts with PIDP. These findings indicate that desvenlafaxine has a favorable tolerability profile, consistent with previous studies on desvenlafaxine in diabetic peripheral neuropathy [21] and major depressive disorder [17]. Consequently, desvenlafaxine appears to be relatively safe for the treatment of PIDP. However, these safety findings should be interpreted with caution. Adverse events in the present study were retrospectively collected from routine clinical records rather than prospectively assessed using a standardized severity grading system. In addition, the exact timing of each adverse event and its relationship to dose adjustment were not systematically documented. Moreover, our study only reported side effects within the dosage range of 50–150 mg, and further research is required to evaluate the potential side effects at higher dosages. It was reported that the metabolism and distribution of desvenlafaxine are not appreciably influenced by variations in cytochrome P450 enzymes or permeability glycoprotein. Because desvenlafaxine’s metabolism is relatively independent of hepatic function, no dosage adjustment is required for patients with hepatic impairment. This contrasts with venlafaxine, which requires dosage reduction in such patients, and duloxetine, whose use is not recommended in case of hepatic insufficiency [25]. In our study, no significant changes in blood pressure and heart rate were observed, which may be attributed to the small sample size. Nonetheless, consistent monitoring of blood pressure and heart rate is essential, as indicated by previous research [20]. Additionally, it is crucial to assess the patient’s comorbidities and concurrent medications before initiating desvenlafaxine for the treatment of PIDP.

## 5. Limitations

Although this study offers clinically relevant real‐word data, several limitations should be acknowledged. First, as a retrospective observational study without a control group, the observed improvement in pain outcomes cannot be attributed solely to desvenlafaxine, and the potential influence of placebo effects, expectation bias, regression to the mean, and the natural course of symptoms cannot be excluded. In addition, the absence of a comparator group limits the interpretation regarding the efficacy of desvenlafaxine. The relatively high treatment response rate observed in this cohort (87.4%) should therefore be interpreted with caution, as it may have been influenced by the retrospective design and the lack of a placebo‐controlled comparison. Future research should focus on conducting prospective, randomized controlled trials to obtain more definitive results. Second, this was a single‐center study with a limited sample size, which may restrict the generalizability of the findings to broader populations of patients with PIDP. In addition, because patients were recruited from a single tertiary referral center, referral bias cannot be excluded, and the study population may not fully represent the overall clinical spectrum of PIDP. The relatively small sample size and imbalance between the responsive and unresponsive groups may also have reduced statistical power and introduced baseline imbalances. Third, this study only tracked NRS‐11 score reductions within the initial 3 months and observed short‐term adverse effects associated with desvenlafaxine. Therefore, the long‐term efficacy, durability of pain relief, optimal duration of treatment, and long‐term safety profile of desvenlafaxine in PIDP remain uncertain. Additionally, although patients with a history of psychiatric illness or substance abuse and those with other concomitant painful medical conditions were excluded, potential confounding factors were not fully evaluated. In particular, psychological symptoms, pain catastrophizing, and other pain‐related psychosocial factors were not systematically assessed using standardized instruments. As a result, residual confounding may still have influenced pain perception, treatment response, and patient‐reported outcomes. Finally, in this study, the cohort of 103 patients with PIDP consisted predominantly of middle‐aged and older women (74.8%) with a mean age of 50.1 years. These observations align with the widely accepted notion that PIDP tends to exhibit a notable prevalence among the female population [6, 8, 14, 15, 26]. The efficacy of desvenlafaxine in male patients and in other age groups cannot be readily extrapolated from the present findings. Further large‐scale, multicenter, prospective studies are warranted to validate these findings.

## 6. Conclusions

Oral administration of desvenlafaxine can serve as an effective and safe therapeutic approach for patients with PIDP. Patients with a short history of PIDP are more likely to benefit from desvenlafaxine treatment. Our results may provide a future standard treatment protocol for PIDP based on clinical evidence.

**Nomenclature**
PIDP: Persistent idiopathic dentoalveolar pain
ICOP: International Classification of Orofacial Pain
TCAs: Tricyclic antidepressants
SNRIs: Serotonin–norepinephrine reuptake inhibitors
NRS‐11: Numeric Rating Scale‐11
HDRS: Hamilton Depression Rating Scale

## Author Contributions

All authors made a significant contribution to the work reported, whether that is in the conception, study design, execution, acquisition of data, analysis, and interpretation, or in all of these areas; took part in drafting, revising, or critically reviewing the article; and have agreed on the journal to which the article has been submitted. This retrospective study was designed by Fang Luo.

## Funding

This study was supported by the National Key Research and Development Program of China (Grant nos. 2022YFC3602200, 2022YFC3602201, 2022YFC3602202, 2022YFC3602203, and 2022YFC3602205), the Capital’s Funds for Health Improvement and Research (Grant no. 2022‐1‐4061), and the National Health Commission Capacity Building and Continuing Education Center Cancer Pain Management (Grant no. PMT1001‐1).

## Disclosure

All authors gave final approval of the version to be published and agreed to be accountable for all aspects of the work.

The sponsor had no role in the trial design, trial conduct, data handling, data analysis, or writing and publication of the manuscript.

## Ethics Statement

This retrospective, single‐center observational study was performed at Beijing Tiantan Hospital, Capital Medical University, adhering to the principles of the Declaration of Helsinki. The study was approved by the Institutional Review Board of the Beijing Tiantan Hospital (Approval Number: KY2026‐022‐02).

## Consent

Informed consent was obtained from all patients before inclusion in the study, and each participant was thoroughly informed that their involvement would be entirely voluntary and that they retained the right to withdraw from the study at any time.

## Conflicts of Interest

The authors declare no conflicts of interest.

## Data Availability

The data that support the findings of this study are available on request from the corresponding author. The data are not publicly available due to privacy or ethical restrictions.
